# Investigating lactoferrin and somatic cell count dynamics in early postpartum Shami and Baladi goats

**DOI:** 10.3389/fvets.2025.1625434

**Published:** 2025-07-04

**Authors:** Montaser Elsayed Ali, Mohamed Abdelrahman, Asem Mohammed Zakaria, Belal Farrag Farag, Ragab Hassan Mohamed, Hayat Fayed, Ibrahim Samir Abd El-Hamid, Fatma Ali, Rabee M. Gheetas, Safaa Y. Nour, Khalid M. Alsyaad, Min Gao

**Affiliations:** ^1^Department of Animal Productions, Faculty of Agriculture, Al-Azhar University, Assiut, Egypt; ^2^Animal Production Department, Faculty of Agriculture, Assuit University, Asyut, Egypt; ^3^Department of Food Hygiene, Faculty of Veterinary Medicine, Aswan University, Aswan, Egypt; ^4^Department of Theriogenology, Faculty of Veterinary Medicine, Aswan University, Aswan, Egypt; ^5^Department of Animal Medicine (Internal Medicine), Faculty of Veterinary Medicine, Benha University, Benha, Egypt; ^6^Department of Animal Physiology, Animal and Poultry Production Division, Desert Research Centre, Cairo, Egypt; ^7^Physiology Department, Faculty of Veterinary Medicine, Aswan University, Aswan, Egypt; ^8^Animal Production Faculty of Agriculture, Al-Azhar University, Cairo, Egypt; ^9^Animal Medicine Department, Faculty of Veterinary Medicine, Aswan University, Aswan, Egypt; ^10^Department of Biology, College of Science, King Khalid University, Abha, Saudi Arabia; ^11^State Key Laboratory of Reproductive Regulation & Breeding of Grassland Livestock, Inner Mongolia University, Hohhot, China; ^12^National Sheep Genetic Evaluation Centre, Inner Mongolia University, Hohhot, China

**Keywords:** lactoferrin, somatic cell count, hematological, follicular dynamics, goat

## Abstract

Earlier studies found that milk lactoferrin (LF) concentration was significantly associated with somatic cell count (SCC) dynamics and lactation stage was related to milk production and mammary gland health, which helps determine the physiopathological conditions and goats’ postpartum activity. The presented study aimed to design an integrated monitoring system among LF and SCC dynamics and hematological indices over the postpartum timeline in Baladi and Shami goats. After collecting the data, the Shami and Baladi groups were compared using independent t-tests, and Pearson’s correlation analysis was performed between variables. This study reported that Shami and Baladi goats’ LF concentrations and SCC had fallen incrementally from day 1 to 35 days postpartum (DPP), and both LF and SCC showed (*p* < 0.05) correlation in early DPP. However, Baladi goats were significantly higher (*p* < 0.01) in milk yield, fat percentage, and protein at 15 DPP than Shami goats. Also,no significant differences (*p* > 0.05) were found in salts, solids, not fat, and total solids between the two groups at 35 DPP. However, total protein, albumin, and globulin-related parameters were significantly higher (*p* < 0.01) at 15 DPP in Baladi compared to Shami goats. The Baladi goats were significantly higher for energy-related parameters and total protein and globulin-related parameters at 35 DPP. As expected, some correlation (*p* < 0.05) between LF and SCC was also seen during early DPP. Furthermore, Shami goats lymphocytes (LYM) × 10^3^/μL, granulocytes (GRA), hemoglobin (HGB), and mean corpuscular hemoglobin concentration (MCHC) were significantly higher (*p* < 0.05) at 15 DPP. Also, at 35 DPP, white blood cells (WBC), lymphocytes (LYM), and monocytes (MONO) were lower in the Shami goats. In conclusion, this study provides essential physiological benchmarks for tracking goat lactation recovery by showing distinctive patterns of lactoferrin and somatic cell count dynamics and hematological adaptation during the postpartum period.

## Introduction

Goats are a significant livestock species in Egypt, substantially affecting the livestock production economy, making goats influential contributors to ruminant production, as they can thrive in highly harsh climates and arid systems (1). As the main local goat breeds, the Shami goat differs from the Baladi goat, and each possesses distinct production characteristics. While the Shami goat originated in Syria and Lebanon and is characterized by its large build, convex nose, long, drooping ears, and, in most cases, the absence of horns (2), conversely, the Baladi goat is a medium-sized, indigenous breed widespread throughout the Middle East and North Africa, including Egypt, Palestine, and Jordan, it is found in various colors and can be horned (3). Regarding productivity, the Shami goat produces approximately 4 liters of milk daily, while the Baladi goat produces only 1 to 3 liters daily. The Shami goat also grows faster and produces more meat (4).

On the other hand, the Baladi goat needs less requirement and has better adaptation to harsh environments (5, 6). Shami goats often give birth to twins, allowing for greater litter numbers, while baladi goats have a lower twin rate. This promoted Shami goats suitable for intensive rearing, while baladi goats are traditionally raised for their ability to withstand harsh conditions (7).

Shami goats experience 210–240 days of lactation, with their peak milk production occurring between 4 and 8 weeks postpartum. Shami goats exhibit a high milk yield and lactation length, paid for by most local goat breeds (8). Baladi goats have a shorter lactation period, and various factors can influence lactation performance, including breed, genetics, availability of feed, and management practices (9). Variability of lactation performance due to breed genetics, availability of feed, and management practices between Shami goats and Baladi goats may contribute to variability (5).

For dairy ruminants, postpartum physiological changes, especially blood parameters, are closely related to future milk production and quality. Blood offers relevant markers for assessing health status (10), pregnancy and postpartum-associated metabolic changes (11), and pregnancy and postpartum-associated metabolic changes (12). Proper management and nutritional interventions to address negative energy balance, reduce oxidative stress, enhance immune function, and regulate hormonal balance can help optimize blood parameters, improve milk yield, and maintain somatic cell count (13). Also, goat milk’s immunological and nutritional aspects are especially critical for kids’ growth, and it has various proteins in milk that have protective functions and significant metabolic roles. Immunoglobulins provide non-specific immune defense, while other proteins such as lysozyme, lactoperoxidase, and lactoferrin deliver targeted, antimicrobial protection (14). Also, lactoferrin (LF) is a multifunctional glycoprotein belonging to the transferrin family, as it can be found in mammalian milk, leukocytes, and various body exocrine secretions (15). Moreover, LF was reported to have multiple bioactive actions, such as serving as a control factor stimulating cellular growth or regulating innate immune defenses (16, 17). So, LF is known to follow a specific temporal model with concentration, increasing concentration with the first colostrum and decreasing during the second half of lactation (18). Moreover, LF greatly impacts the non-specific host defenses in the mammary and other epithelial tissues by being incorporated into antimicrobial peptide structures (19). The two mechanisms that mediate its bacteriostatic and bactericidal activities are (i) iron impounding, which creates conditions for inhibiting microbial growth, by (ii) directly disrupting the metabolic activity for active bacteria’s membranes.

Goat milk contains a somatic cellular composition consisting of mammary epithelial cells (60–80%) and leukocytes (20–40%), which include macrophages, neutrophils, and lymphocytes (20). These cells are integral in the turnover and repair of mammary tissues, in localized immune defense surveillance, and in active body protection against invasion. While somatic cell counts (SCC) tend to vary cyclically (generally <1 × 10^6^ cells/mL in goats), increased values indicate subclinical mastitis, some degree of epithelial injury, and a change in milk yield (21). Hence, the most fundamental factors controlling SCC variation are (1) stage of lactation (higher in early/late lactation), (2) differences among breeds (dairy > meat breeds), and (3) milking management (22).

This study investigates the postpartum physiological and lactation dynamics to forge cohesive monitoring of postpartum lactation progress in goats, highlighting the physiological changes and their correlation to lactation performance in this critical period.

## Materials and methods

### Animals and management

Forty female goats were employed in this investigation. In the same barn, the goats were divided into two experimental groups, 20 animals in each group: (i) Shami goats were 26 ± 1.25 months of age, with a mean body weight of 25 ± 0.69 kg (mean ± SD), and (ii) Baladi goats were 21 ± 1.39 months of age, with a mean body weight of 23 ± 1.12 kg (mean ± SD). The study was conducted on a private farm in the Aswan Governorate of Egypt (geographic location: 24° 5′20′′ N, 32° 53′59′′ E), 900 km south of Cairo on the east bank of the Nile River. The research was conducted in 2024, and they approved the participation of all animal owners before being included.

The goats were provided for daytime grazing on the farm and housed in a semi-open barn at night. They were also provided with a daily ration of farm feed formulated based on National Research Council nutritional guidelines (23); the chemical composition of the experimental diet consisted of 89.75% dry matter, 85.52% organic matter, 14.15% crude proteins, 53.55% neutral detergent fiber, 20.12% acid detergent fiber, 5.15% ether extract, and 4.23% ash. The animals were all put through a standardized clinical exam to determine their health status, which included heart rate, rectal temperature, ruminal motility, respiratory rate, pulse rate, and capillary refill time (24).

### Milk samples analyzing

#### Accumulate samples

Following rigorous adherence to established protocols to ensure sterility and hygiene, the samples were taken during morning milking sessions on days 1, 15, and 35 DPP. The udder and teats were thoroughly cleaned with warm water and soap after the animal was safely stabilized and then thoroughly disinfected with a 70% alcohol solution (25). Initial milk (approximately 2–3 mL) was discarded to reduce bacterial contamination (26), and samples were then collected in dry, sterile 15 mL tubes, with care taken to prevent any contact with potential contamination sources (27). For transportation to the laboratory, samples were promptly kept in a refrigerator at 4°C, or if the analysis was postponed for more than 24 h, they were frozen at −20°C (28). Every sample was assigned a number, and information about the animal, the time and date of collection, and any remarks about the udder’s health or the milk’s properties were noted. The use of sterile gloves and hand sanitization both before and after collection were among the complete biosafety requirements that were fulfilled.

#### Lactoferrin quantitation

Milk lactoferrin concentrations were measured using a commercial Caprine Lactoferrin ELISA Kit (Catalog No. E11-126, Bethyl Laboratories, United States) according to the manufacturer’s instructions and the method outlined by Chen et al. (29). The average recovery ranged from 92 to 101%, and the intra-assay and inter-assay coefficients of variation (%CV) were less than 10%. Briefly: Milk samples were centrifuged at 3,000 × g for 15 min at 4°C to remove fat. The aqueous phase was diluted 1:100 in PBS (pH 7.4). Standards and samples were incubated in antibody-coated wells for 60 min. After washing, an HRP-conjugated detection antibody was added. Color development was measured at 450 nm using a microplate reader. Concentrations were calculated from the standard curve and expressed in μg/ml.

#### Milk somatic cell count determination

Somatic cell counts were determined with an automated somatic cell counter (Fossomatic™ series, Foss Analytical, Denmark) using the standard procedure of Khalafallah et al. (30). The instrument was calibrated daily using certified reference material, and every sample was measured in duplicate to ensure measurement precision. The results are expressed as milk cells per milliliter (cells/mL).

### Hematological analysis

#### Sample collection

Whole blood samples were collected via jugular venipuncture into vacutainer EDTA-coated tubes (Becton Dickinson, United States) on day 25 and day 35 DPP. Immediately after collection, the samples were put on ice and transported to the laboratory for processing. Strict aseptic practices were adhered to during the collection of the samples to ensure integrity and contamination prevention.

#### Blood biochemicals

Glucose and urea were tested using laboratory kits provided by the Diamond Chemical Company in Germany (31). Total protein using specific kits developed by Spinreact Company, Spain (32). Spectrum Company’s kit (31) used a serum albumin test. Additionally, globulin levels were determined mathematically by subtracting albumin values from total serum protein values. To determine glucose concentrations, sodium fluoride-coated collection tubes were used. The levels of aspartate transaminase (AST) and alanine transaminase (ALT) were determined using assay kits given by Spectrum Chemical Company, Egypt, following the Young methodology (32).

#### Complete blood count analysis

Hematological parameters were analyzed using SPINREACT kits of Chemical Company Girona, Spain (12). The parameters included the following: White blood cells (WBCs): Lymphocytes (LYM; %), Monocytes (MONO; %), and Granulocytes (GRA; %). Red blood cell (RBC) count (×10^6^/μL), Hemoglobin concentration (HGB, g/dL), Packed cell volume (PCV, %), Mean corpuscular volume (MCV, FL), Mean corpuscular hemoglobin concentration (MCH, pg.), Mean corpuscular hemoglobin concentration (MCHC, g/dL). The analyzer was previously calibrated with commercial controls for analysis, and the sample integrity was verified to achieve valid results. The measurements were repeated twice, and the outliers were reverified to ensure compliance.

### Statistical analysis

Data were analyzed in Statistical Package for the Social Sciences (SPSS) 25 after normality checking (Kolmogorov–Smirnov test). Shami and Baladi groups were compared through independent t-tests, while Pearson’s correlation (95% CI) measured correlations between variables.

## Results

### Lactoferrin level (μg/ml) at early lactation

**Figure 1:**
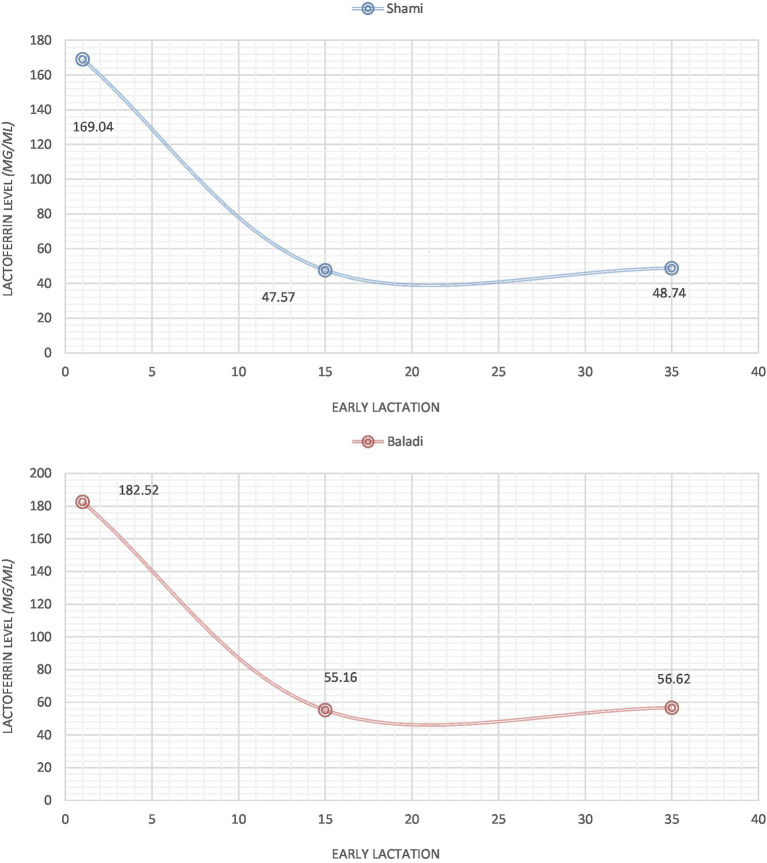
Lactoferrin level (μg/ml) during postpartum early lactation in Shami and Baladi goats.

Figure 1 illustrates the lactoferrin concentration profiles of early lactation in Baladi and Shami goats. The initial measurements at 1 DPP indicated higher lactoferrin concentration profiles in Baladi goats (182.52 μg/mL) compared to Shami goats (169.04 μg/mL) but only slightly so (*p* < 0.05). There was a drastic reduction in both breeds’ lactoferrin levels by 15 days postpartum (Shami: 47.57 μg/mL; Baladi: 55.16 μg/mL; *p* > 0.05), and the levels were comparatively stable across 35 days postpartum (Shami: 48.74 μg/mL; Baladi: 56.62 μg/mL; *p* > 0.05). No significant breed differences at any time point were detected using statistical analysis (*p* > 0.05) despite the consistent numerical trend of Baladi goats having higher lactoferrin levels across the study period.

### Somatic cell count (SCC; cells/ml) for the period of early lactation

**Figure 2:**
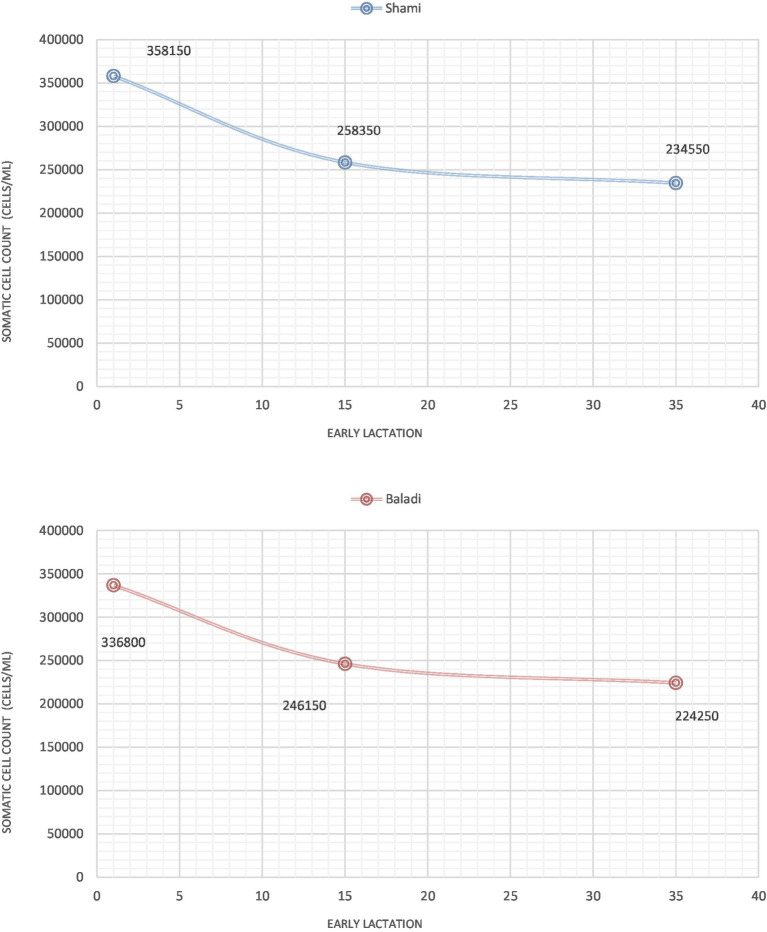
Somatic cell count (SCC; cells/ml), during early lactation as an indicators of postpartum lactation dynamics in Shami and Baladi goats.

As Figure 2 shows the trends for SCC in Shami and Baladi goats through early lactation. On the first day of postpartum, Shami goats had SCC values that were numerically higher than those of Baladi goats, with Shami at (358,150 cells/mL) and Baladi at (336,800 cells/mL) but were not statistically significant (*p* = 0.179). Both breeds exhibited a decline in SCC Shami at 258,350 cells/mL and Baladi at 246,150 cells/mL by the 15-day mark postpartum; also, there was a continued decline in both breeds by the 35-day for Shami goats at 234,550 cells/mL and Baladi goats at 224,250 cells/mL; however, these values were statistically insignificant (*p* = 0.244). Statistical analysis confirmed no significant breed-based variations at any time point (*p* > 0.05), which means both breeds had similar SCC trends regardless of the postpartum period.

### Lactoferrin level (μg/ml) and somatic cell counts (SCC; cells/ml) relation

The correlation analyses of Lactoferrin level and Somatic cell count (SCC) data for Shami and Baladi Goats were integrated in Tables 1, 2.

**Table 1:** Correlation analyses between lactoferrin level and somatic cell count (SCC), in Shami goats.

Item	Somatic cell count (SCC)		
	1 DPP	15 DPP	35 DPP
Lactoferrin level			
1 DPP			
r	−0.320	−0.299	−0.325
P	0.169	0.201	0.162
15 DPP			
r	−0.214	−0.234	−0.186
P	0.365	0.32	0.434
35 DPP			
r	−0.355	−0.382	−0.344
P	0.125	0.097	0.138

DPP, Days postpartum. P, Propapility. r, Correlation value.

**Table 2:** Correlation analyses between lactoferrin level and somatic cell count (SCC), in Baladi goats.

Item	Somatic cell count (SCC)		
	1 DPP	15 DPP	35 DPP
Lactoferrin level			
1 DPP			
r	−0.300	−0.305	−0.161
P	0.199	0.191	0.499
15 DPP			
r	−0.228	−0.193	−0.160
P	0.334	0.414	0.501
35 DPP			
r	−0.310	−0.238	−0.309
P	0.184	0.313	0.185

DPP, Days postpartum. P, Propapility. r, Correlation value.

Lactoferrin levels and somatic cell counts (SCC) in Shami and Baladi goats at 1, 15, and 35 DPP were inversely related, but not statistically significant (*p* > 0.05). In the Shami goats, there was the strongest inverse relationship (*r* = −0.382, *p* = 0.097) at 15 DPP, followed by 1 DPP (*r* = −0.320, *p* = 0.169) and 35 DPP (*r* = −0.355, *p* = 0.125). In the Baladi goats, there were stronger negative correlations at 1 DPP (*r* = −0.305, *p* = 0.191) and 35 DPP (*r* = −0.310, *p* = 0.184), while there were weaker negative correlations at 15 DPP (*r* = −0.193 to −0.238).

### Milk yield and composition

The milk yield and composition in Shami and Baladi Goats During Postpartum Recovery are presented in Table 3. The Baladi goats revealed a significantly higher (*p* < 0.01) milk yield (kg/day), fat %, and protein at 15 DPP compared to the Shami goats. Also, there was significantly higher (*p* < 0.05) lactose % at 15 DPP in Baladi goats. On the other hand, no significant differences (*p* > 0.05) were observed in salt content, solids not fat, and total solids content between Shami and Baladi goats at 15 DPP. At the same time, there was a significance (*p* < 0.05) higher in the Shami goats than Baladi goats in solids % at 35 DPP. Furthermore, at 35 DPP, the Baladi goats showed a significant increase in milk yield (kg/day), fat %, and protein compared to the Shami goats. However, no significant differences (*p* > 0.05) were observed in the Salts %, Lactose % solids not fat %, and Total solids % between Shami and Baladi goats at 35 DPP.

**Table 3:** Milk yield and composition in Shami and Baladi goats during postpartum recovery.

Days postpartum (DPP)	Parameter	Shami	Baladi	SEM	Sig	*p*-value
15 DPP	Milk yield (kg/day)	0.43	0.56	0.02	**	0.001
	Fat %	3.05	3.27	0.05	**	0.029
	Protein %	2.97	3.14	0.03	**	0.010
	Salts %	0.72	0.71	0.00	Ns	0.013
	Lactose %	4.80	5.06	0.07	*	0.050
	Solids not fat%	8.29	8.48	0.08	Ns	0.286
	Total solids %	11.76	11.77	0.08	Ns	0.985
35 DPP	Milk yield (kg/day)	1.80	1.63	0.20	**	0.008
	Fat %	3.14	3.42	0.06	*	0.018
	Protein %	3.03	3.49	0.08	**	0.002
	Salts %	0.71	0.61	0.28	*	0.032
	Lactose %	4.55	4.72	0.05	Ns	0.093
	solids not fat %	8.52	8.55	0.07	Ns	0.861
	Total solids %	11.84	11.87	0.11	Ns	0.904

DPP, Days postpartum. Ns, non significance. *Significant at the 0.05. **Significant at the 0.01.

### Blood biochemical

The blood biochemicals in Shami and Baladi Goats During Postpartum Recovery are presented in Table 4. For the total protein, albumin, and globulin-related parameters (mg/dl), the Bladi goats were significantly higher (*p* < 0.01) at 15 DPP compared to Shami goats. While energy-related parameters (mg/dl), the glucose concentrations did not differ significantly between goats at 15 DPP. Also, no significant differences (*p* > 0.05) were observed in the liver function enzymes (ALT and AST) and urea (mg/dl) at 15 and/or 35 DPP between groups. Furthermore, at 35 DPP, the energy-related parameters (mg/dl) and the glucose concentrations were significantly higher (*p* < 0.01) in the Baladi compared to the Shami goats. For the total protein and globulin-related parameters (mg/dl), the Bladi goats were significantly higher (*p* < 0.01) at 35 DPP compared to Shami goats.

**Table 4:** Blood biochemical in Shami and Baladi goats during postpartum lactation recovery.

Days postpartum (DPP)	Parameter	Shami	Baladi	SEM	Sig	*p*-value
15 DPP	Glucose (mg/dl)	105.45	107.35	1.30	Ns	0.473
	Total protein (mg/dl)	6.31	6.86	0.08	**	0.001
	Albumin (mg/dl)	2.87	3.30	0.07	**	0.004
	Globulin (mg/dl)	2.99	3.45	0.09	**	0.010
	Alanine transaminase (mg/dl)	18.49	18.56	0.25	Ns	0.893
	Aspartate transaminase (mg/dl)	33.24	36.29	0.88	Ns	0.086
	Urea (mg/dl)	21.28	21.65	0.23	Ns	0.435
35 DPP	Glucose (mg/dl)	102.50	114.07	1.69	**	0.001
	Total protein (mg/dl)	6.87	7.49	0.13	*	0.015
	Albumin (mg/dl)	3.02	3.21	0.08	Ns	0.207
	Globulin (mg/dl)	3.20	3.51	0.07	*	0.022
	Alanine transaminase (mg/dl)	19.07	20.14	0.25	Ns	0.059
	Aspartate transaminase (mg/dl)	34.04	37.25	0.82	Ns	0.052
	Urea (mg/dl)	21.55	24.35	0.42	Ns	0.085

DPP, Days postpartum. Ns, non significance. *Significant at the 0.05. **Significant at the 0.01.

### Complete blood count parameters

Table 5 displays the hematological comparison of Shami and Baladi goats during the postpartum. Shami goats had slightly higher lymphocyte counts (10^3^/μL; *p* = 0.050) and higher granulocyte counts (10^3^/μL; *p* = 0.040) at 15 days postpartum (DPP). Alongside these variations in white blood cell parameters, Shami goats showed superior red blood cell indices, such as a significantly higher mean corpuscular hemoglobin concentration (g/dL; *p* = 0.039) and hemoglobin concentration (g/dL; *p* = 0.040). The leukocyte profile also revealed additional breed differentiation at 35 DPP, with Shami goats exhibiting lower monocyte levels (10^3^/μL; *p* = 0.042) and significantly higher total white blood cell counts (10^3^/μL; *p* = 0.020) and lymphocyte numbers (10^3^/μL; *p* = 0.003). By this point, the Shami goats’ initial granulocyte advantage had faded, and both breeds displayed similar levels (10^3^/μL; *p* = 0.171). Although the early breed-specific variations in hemoglobin and MCHC were no longer statistically significant at 35 DPP, red blood cell parameters stayed constant throughout the study. The patterns showed that as lactation continued, both breeds’ granulocyte concentrations decreased while their overall white blood cell counts increased, indicating dynamic physiological adaptations during the postpartum phase.

**Table 5:** Complete blood count parameters in Shami and Baladi goats during postpartum recovery.

Days postpartum (DPP)	Parameter	Shami	Baladi	SEM	Sig	*p*-value
15 DPP	WBC (×10^3^/μL)	19.69	19.29	0.12	Ns	0.110
	LYM (×10^3^/μL)	4.77	4.55	0.06	*	0.050
	MONO (×10^3^/μL)	1.79	1.60	0.06	Ns	0.930
	GRA (×10^3^/μL)	13.55	13.23	0.08	*	0.040
	RBC (×10^6^/μL)	12.02	11.90	0.16	Ns	0.700
	PCV (%)	20.02	19.23	0.21	Ns	0.060
	HGB (g/dL)	8.33	7.93	0.10	*	0.040
	MCV (FL)	18.26	17.86	0.11	Ns	0.060
	MCHC (g/dL)	39.16	38.31	0.21	*	0.039
	MCH (pg.)	7.54	7.16	0.11	Ns	0.073
35 DPP	WBC (×10^3^/μL)	22.33	23.96	0.14	*	0.020
	LYM (×10^3^/μL)	4.48	5.18	0.12	*	0.003
	MONO (×10^3^/μL)	0.91	1.09	0.04	*	0.042
	GRA (×10^3^/μL)	6.10	5.70	0.15	Ns	0.171
	RBC (×10^6^/μL)	12.71	12.44	0.14	Ns	0.332
	PCV (%)	20.99	20.54	0.34	Ns	0.522
	HGB (g/dL)	8.29	8.31	0.13	Ns	0.928
	MCV (FL)	17.98	17.68	0.13	Ns	0.275
	MCHC (g/dL)	39.68	39.46	0.26	Ns	0.686
	MCH (pg.)	7.40	7.19	0.09	Ns	0.238

DPP, Days postpartum. Ns, non significance. *Significant at the 0.05. **Significant at the 0.01. WBC, White blood cells; LYM, Lymphocytes; MONO, Monocytes; GRA, Granulocytes; RBC, Red blood cells; PCV, Packed cell volume; HGB, Hemoglobin; MCV, Mean corpuscular volume; MCHC, Mean corpuscular hemoglobin concentration; MCH, Mean corpuscular hemoglobin.

## Discussion

Small ruminants’ immune status, which depends on complex interactions with their productivity and physiological state, influences their postpartum lactation performance. While several previous studies have shown a close correlation between milk lactoferrin (LF) concentration and somatic cell count (SCC) dynamics and that the lactation stage is associated with milk production and mammary gland health, it can help identify pathophysiological conditions and postpartum activity in goats. This study successfully designed an integrated monitoring system for somatic cell and red blood cell count dynamics and hematological parameters over the postpartum period in Baladi and Shami goats.

This study showed that lactoferrin concentrations from Baladi goats were significantly higher at 1 day postpartum (DPP) than those of Shami goats. However, for the goats below 35 DPP (where lactation progresses), the amount of lactoferrin in both types was significantly lower. The results were consistent with the findings of Navarro et al. (34), who determined that the lactoferrin (LF) in ovine colostrum significantly decreased after day two of milking and similar to those reported in goat milk, according to Hiss et al. (35) and Chen et al. (36).

However, this study found that at 1 DPP, somatic cell counts (SCC) were slightly higher in Shami goats than in Baladi goats. From there, they gradually decreased in both breeds to 35 DPP. These results are consistent with physiological changes expected in the postpartum phase, where SCC levels typically decline as lactation progresses past the colostral phase (37, 38). However, according to studies by Suzuki et al., milk’s lactoferrin content is greater when drying off than postpartum. The positive link between postpartum milk SCC and lactoferrin concentration suggests that, under pathological simulation, immune cells infiltrate the udder more often, and the lactoferrin these cells produce elevates the lactoferrin concentration in milk (39). Also, previous goat results indicated that SCC is typically greater in less productive, healthy animals. On the other hand, among milk controls, goats that produce more than 3 kg of milk per day had the lowest SCC (<954 × 10^3^ cells/mL), which is probably caused by damaged mammary gland alveolar cells, which raises milk SCC significantly (40).

The present study reported a consistent negative correlation between lactoferrin concentrations and somatic cell counts (SCC) during early lactation. Elevated lactoferrin levels in early lactation may enhance immune defense, thereby reducing leukocyte recruitment to the mammary gland, a phenomenon reflected in lower SCC values. Additionally, the observed decline in SCC during this phase could signify the resolution of physiological inflammation following parturition, coinciding with the transition from colostrum to milk production (40). Early lactation yields the most milk, which declines, while SCC peaks soon after birth. For example, the SCC in buffaloes’ healthy quarters rises from around 80 × 10^3^ cells/mL at 35 days after giving birth to 160 × 10^3^ cells/mL at 285 days.

This Study reported that the Baladi goats generated consistently larger milk production at both 15 and 35 DPP than the Shami goats, consistent with prior research demonstrating that indigenous breeds perform better in lactation under similar management conditions (41). Baladi goats have greater fat and protein percentages, indicating a genetic inclination to produce more energy-dense milk, which is essential for kid growth and dam recovery (42).

However, there was a significantly higher lactose concentration at 15 DPP in Baladi goats. Depending on the metabolic state, lactose production during the early stages of lactation is impacted, which influences the milk yield (43). The lower SCC and higher Milk yield (kg/day) in Baladi goats than in Shami goats may affect milk lactose. Also, Research shows that goats with high hemoglobin levels produce higher milk and have lower SCC, while goats with low hemoglobin levels (<5.6 mmol) have greater SCC. This implies that in small ruminants, hemoglobin levels may affect milk production and udder health to fight infections, and immune cells from the blood will pass to the udder, raising milk SCC (44).

In the present study, at 15 DPP, the Bladi goats had significantly higher levels of total protein, albumin, and globulin-related parameters than the Shami goats. Additionally, the Aladi goats had significantly higher levels of total protein and globulin-related parameters at 35 DPP than the Shami goats. On the other hand, stable blood globulin levels and healthy immunological activity can help reduce SCC and increase milk production (45).

The CBC profile revealed breed differentiation at 35 DPP, with Shami goats showing lower monocyte levels and higher total white blood cell counts and lymphocyte numbers. There are several possible causes for the high WBC counts in the early stages of lactation: (1) immune activation following delivery to prevent possible infections (46), (2) physiological reactions to the involution of the uterus (47), and (3) leukocyte migration to mammary tissue to increase phagocytic activity and strengthen antimicrobial defenses (48). Also, the primary cell type in the milk of healthy, uninfected goats is polymorphonuclear neutrophils (PMN) PMNs, whereas the predominant cell type in the milk of uninfected sheep is macrophages. When the equilibrium of blood lymphocytes and neutrophils is upset, such as during stress or immunological reactions, their proportions in milk may shift, impacting milk SCC and yield. For example, there is a negative link between blood neutrophil numbers and milk SCC, while there is no significant correlation between lymphocyte counts and SCC (49). However, most hematological parameters notably stayed within homeostatic ranges (50), indicating the resilience of physiological regulation systems.

## Conclusion

This study clarifies important physiological patterns in Shami and Baladi goats during the postpartum phase physiologically correlated with milk yield and composition changes. The current result demonstrates dynamic changes in lactation, somatic cell dynamics, and physiological adaptations. The present results provide physiological evidence for tracking goat health during the critical transition period, providing valuable information for managing and optimizing dairy production performance.

## Data Availability

The raw data supporting the conclusions of this article will be made available by the authors without undue reservation.
